# Supplementary material for: Pharmacological modulation of AMPA receptors rescues specific impairments in social behavior associated with the A350V Iqsec2 mutation
Source: Transl Psychiatry. 2021 Apr 22;11:234. doi: 10.1038/s41398-021-01347-1 (PMC8062516; doi:10.1038/s41398-021-01347-1)
Supplement: Supplementary file 1 — Supplementary figures [file 41398_2021_1347_MOESM1_ESM.pdf]

Supplementary figures:

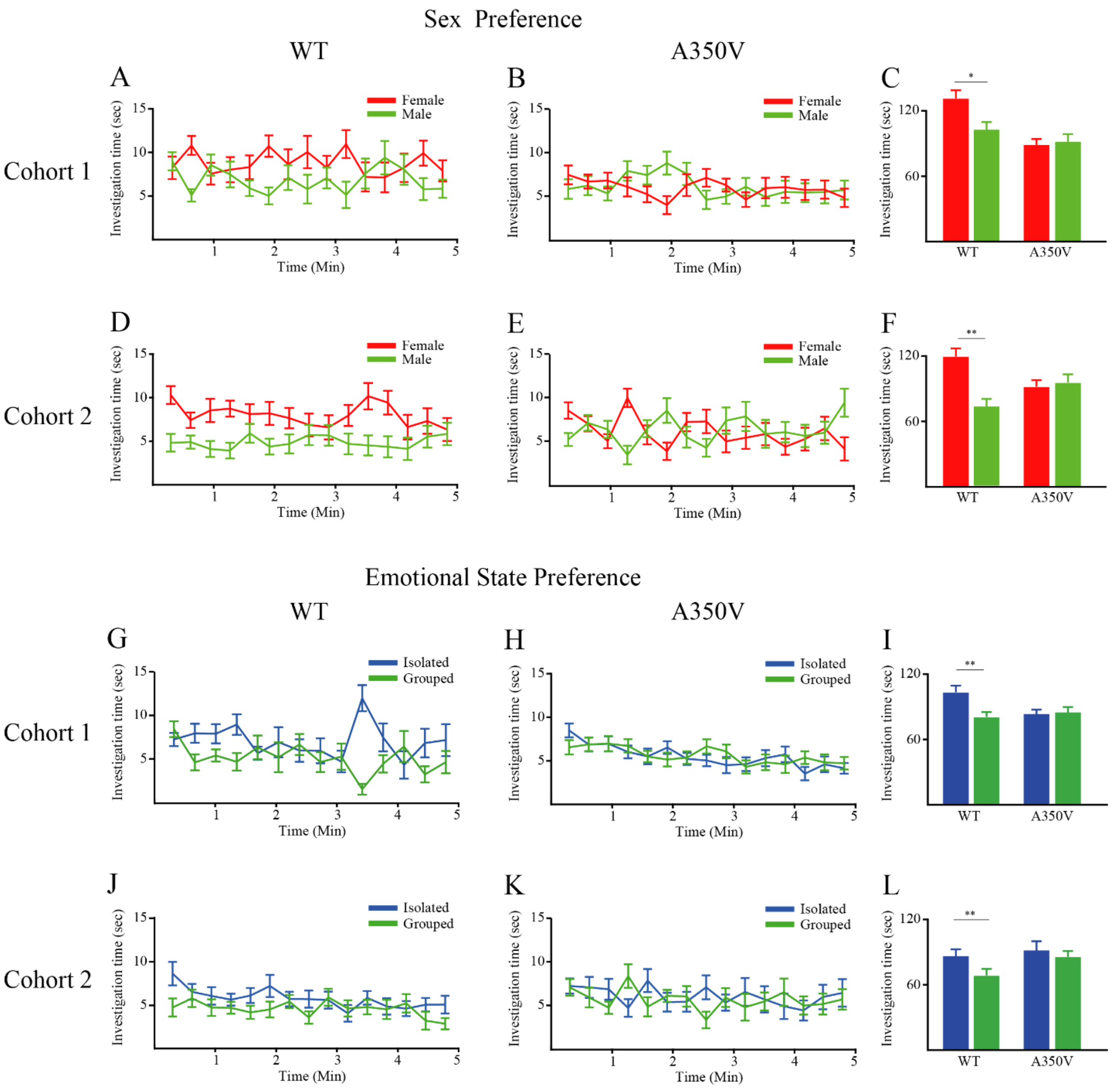

Figure S1. Deficits in sex preference and emotional state preference behaviors are replicated in two cohorts of A350V *Iqsec2* mice

(A-B) Mean investigation time measured separately for each stimulus (20-s bins) across the SxP session for the first cohort of WT (A, n=14) and A350V (B, n=20) mice.

(C) Mean investigation time summed separately for each stimulus throughout the SxP test for WT (left) and A350V (right) subjects. *Post hoc* paired t-tests following main effect in mixed model ANOVA test. WT: t_{13}=2.106, *p<0.05; A350V: t_{19}=-0.274, p=0.787.

(D-F) Same as A-C, for a second cohort of animals (WT. *post hoc* paired t-tests following main effect in mixed model ANOVA test. WT (n=19) t_{18}=3.267; **p<0.01; A350V (n=16) t_{15}=-0.307, p=0.763.

(G-L) Same as A-F, for the ESP test. *post hoc* paired t-tests following main effect in mixed model ANOVA test. I, WT (n=11) t_{10}=4.28; **p<0.01, A350V (n=28): t_{27}=-0.172, p=0.865; L, WT (n=17) t_{16}=3.11; **p<0.01, A350V (n=15) t_{14}=-0.513, p=0.616.

All error bars represent SEM.

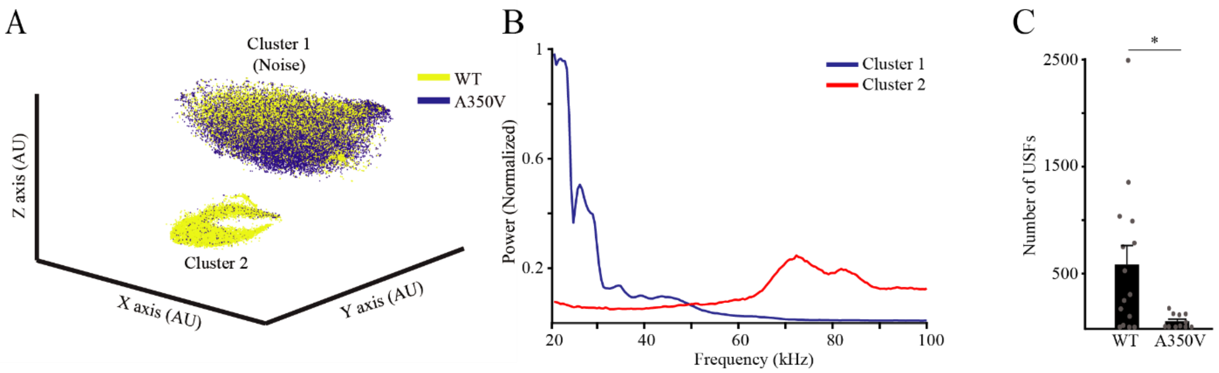

Figure S2. A350V *Iqsec2* male mice do not emit mating calls in the presence of a female mouse – A second cohort.

(A) 3D t-SNE analysis of all ultrasonic fragments emitted by WT (yellow; n=15) and A350V (blue; n=11) adult male mice during an encounter with a female C57BL/6J mouse. Note that while cluster 1 (noise) seem to contain similar number of fragments from both genotypes, cluster 2 (vocalizations) contains almost only fragments from WT mice.

(B) Power Spectral Density (PSD) analysis of all USFs recorded from all animals, calculated separately for each cluster. Note that in contrast to the PSD profile of cluster 1 which shows a wide range of frequencies, mainly at the lower range, the profile of cluster 2 shows a clear peak at the range of 60-90 kHz.

(C) Comparison of the number of USFs from cluster 2 between the two genotypes. Note that A350V mice emitted significantly lower number of USFs as compared to WT animals (Mann-Whitney U test. U=39.000, $*p<0.05$).

All error bars represent SEM.

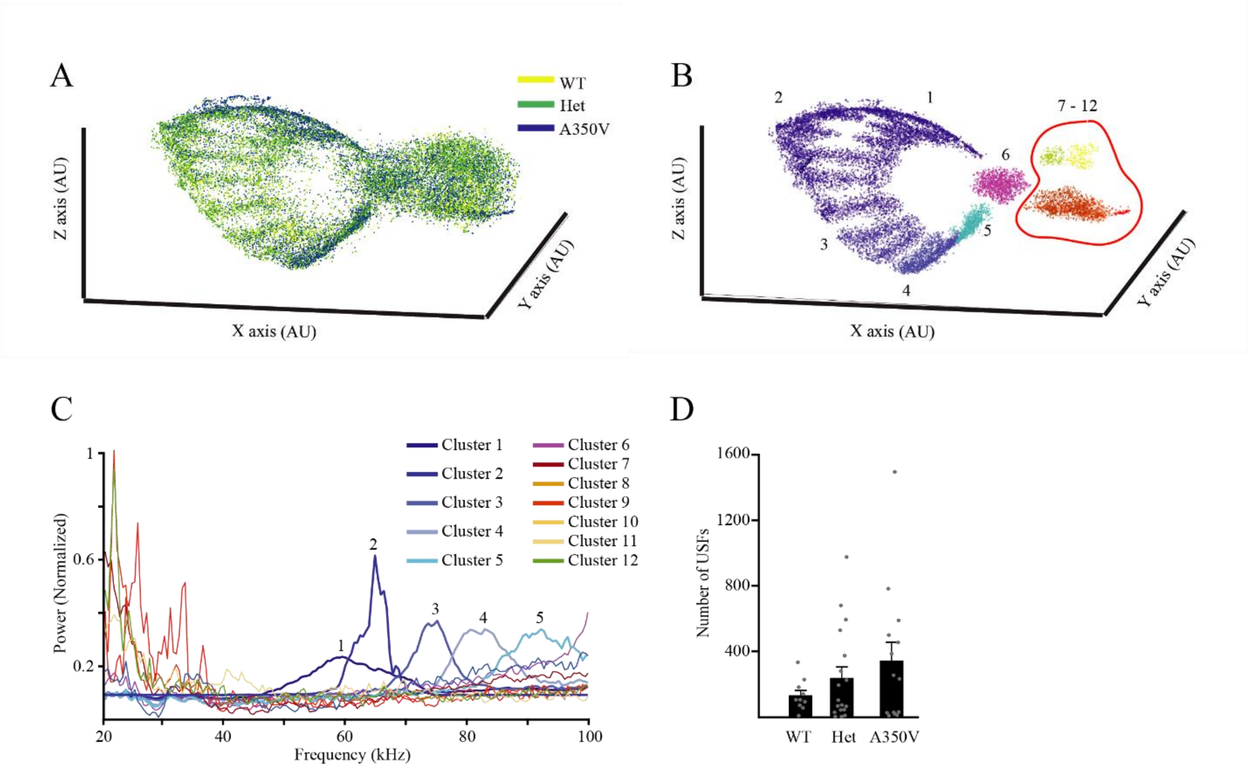

Figure S3. No difference in pup separation calls between WT and A350V *Iqsec2* littermates

(A) 3D t-SNE analysis of all ultrasonic fragments (USFs) recorded during pup separation experiments of WT (yellow; males and females; n=10), heterozygous (green; females; n=18) and A350V (blue; males; n=14) mice. All pups were genotyped after the recordings.

(B) The same t-SNE analysis after automatic clustering (DBSCAN) of the various USFs.

(C) PSD analysis of all USFs from each cluster for all animals analyzed in A. Note the while clusters 6-12 contain fragments of noise, clusters 1-5 contain fragments of genuine vocalizations with distinct peaks in the range of 50-100 kHz.

(D) Comparison of the number of USFs from clusters 1-5 between the three groups of animals analyzed in A. There was no significant difference between the groups (Kruskal Wallis test, Chi=0.469, p=0.79).

All error bars represent SEM.

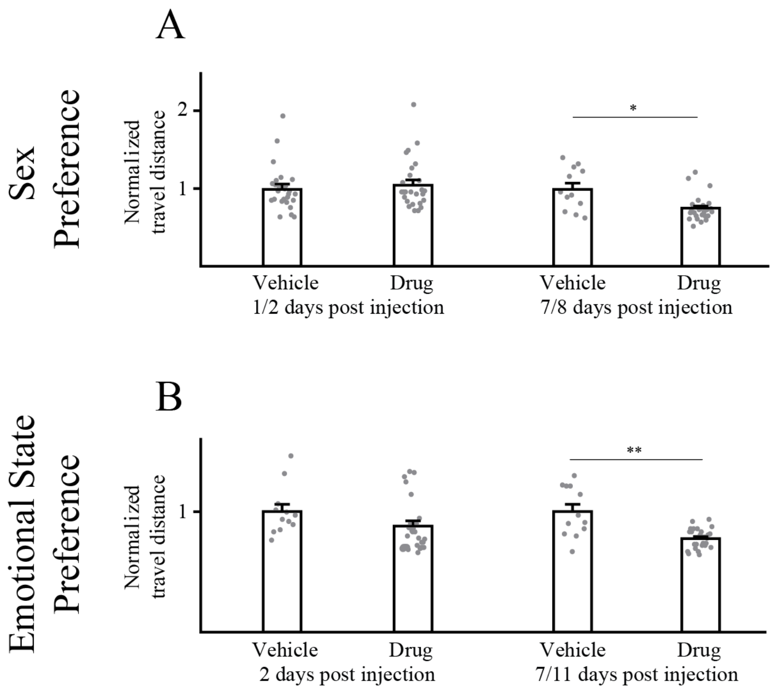

Figure S4. A significant effect of PF-4778574 on the traveled distance 7-11 days after injection

(A) The mean distance traveled by subject A350V mice injected with either vehicle of PF-4778574, during the SxP test conducted either 1-2 days (left bars) or 7-8 days (right bars) post drug administration. The results for each time point were normalized by dividing each data point by the mean of the vehicle injected group.

(B) The mean distance traveled by subject A350V mice injected with either vehicle of PF-4778574, during the ESP test conducted either 1-2 days (left bars) or 7-11 days (right bars) post drug administration. The results for each time point were normalized by dividing each data point by the mean of the vehicle injected group.

*p<0.05, **p<0.01, post-hoc t-test following main effect in ANOVA test. All error bars represent SEM.

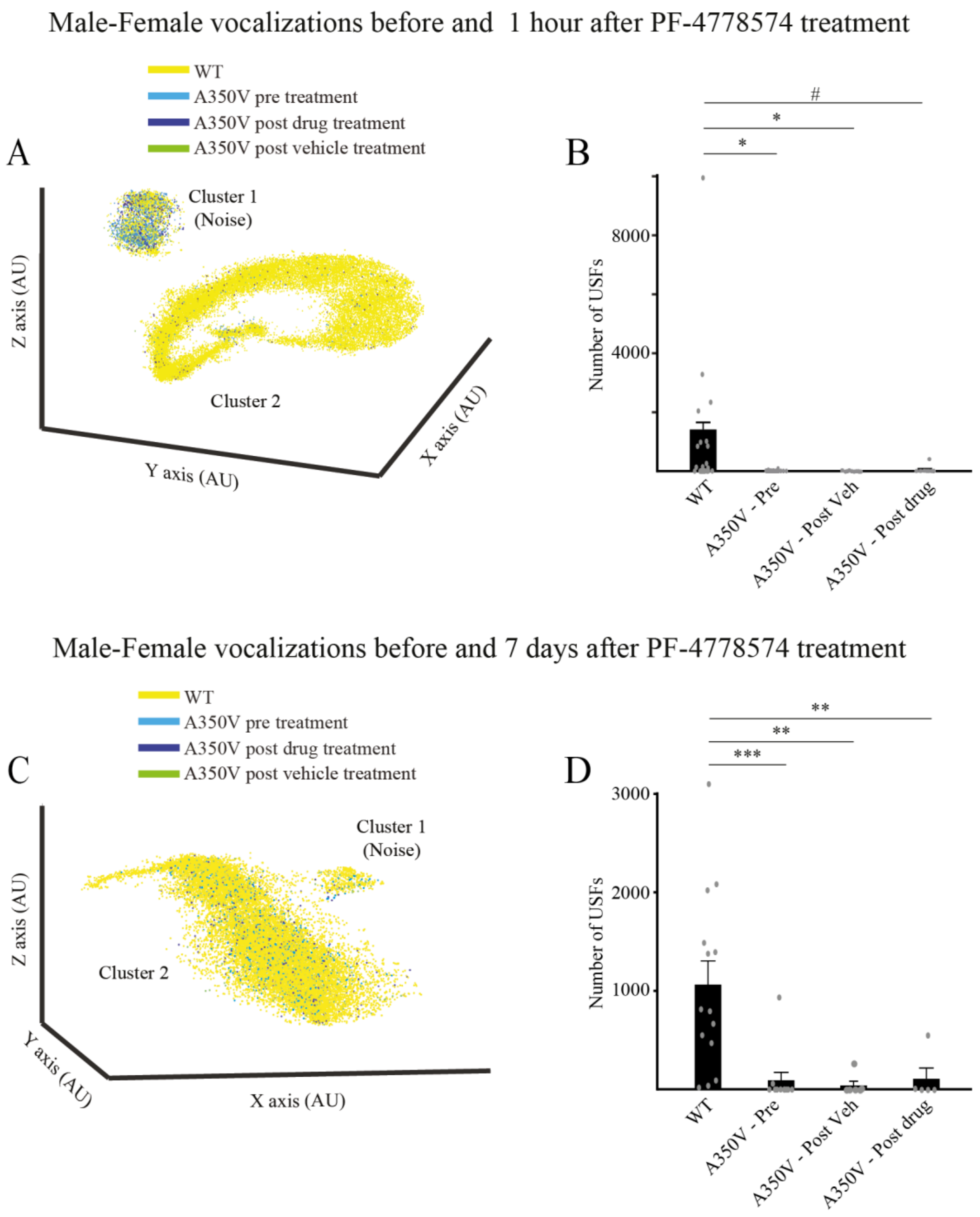

Figure S5. PF-4778574 does not affect the impaired mating calls in A350V *Iqsec2* mice.

(A) 3D t-SNE analysis of all ultrasonic fragments (USFs) recorded during male-female interactions of WT mice (yellow; n=31), as well of A350V mice, before (light blue; n=36) or 1 hour after injection of either PF-4778574 (blue; n=11) or vehicle (green; n=10). Note that while cluster 1 (noise) seem to contain similar number of fragments from both genotypes, cluster 2 (vocalizations) contains almost only fragments from WT mice.

(B) Comparison of the number of USFs from cluster 2 between the four groups of animals analyzed in A. Note that PF-4778574 administration did not elicit any effect on the number of USFs emitted by MT animals (Kruskal Wallis test, chi=8.737, $p=0.033$, post hoc Mann-Whitney U test following main effect: WT-MT before injection – $U=390$, $p<0.05$; WT-MT after PF-4778574 injection – $U=81$, $p<0.05$; WT-MT after vehicle injection – $U=107.5$, $p=0.071$).

(C-D) As in A-B, for different cohorts of animals, with the vocalizations post treatments (PF-4778574 and vehicle injections) recorded 7 days following injections. Here too, no effect of PF-4778574 was observed. (WT - $n=14$, MT before injection - $n=11$, MT after vehicle injection - $n=6$, MT after PF-4778574 injection - $n=5$; Kruskal Wallis test, chi=20.31, $p<0.001$, *post hoc* Mann-Whitney U test following main effect: WT-MT before injection – $U=10$, $p<0.001$; WT-MT after PF-4778574 injection – $U=6$, $p<0.01$; WT-MT after vehicle injection – $U=4$, $p<0.01$).

All error bars represent SEM.
